# Resources utilisation and economic burden of percutaneous vertebroplasty or percutaneous kyphoplasty for treatment of osteoporotic vertebral compression fractures in China: a retrospective claim database study

**DOI:** 10.1186/s12891-020-03279-1

**Published:** 2020-04-17

**Authors:** Dehong Yang, Yanlei Zhang, Xiao Ma, Li Huo, Liran Li, Yue Gao

**Affiliations:** 1 Department of Spinal Surgery, Nanfang Hospital, Southern Medical University, AD: No.1838 North Guangzhou Avenue, Guangzhou, 510515 PR China; 2 Shanghai Branch, Lilly Suzhou Pharmaceutical Co.Ltd, AD: No. 288 Shimen No.1 Road, Jing’an District Shanghai, 200041 PR China; 3 Department of Health Economics, Shanghai Centennial Co. Ltd, AD: 702A, B Block, Fenglin International Center, No. 388 Fenglin Road, Shanghai, 200030 PR China

**Keywords:** OVCF, Percutaneous vertebroplasty, Percutaneous kyphoplasty, Surgery, Costs

## Abstract

**Background:**

Osteoporotic vertebral compression fractures (OVCF) is a common and often debilitating complication of osteoporosis, leading to significant morbidity and increased mortality. Percutaneous vertebroplasty (PVP) and Percutaneous kyphoplasty (PKP) are recommendable surgical treatments for OVCF.

**Objective:**

To evaluate PVP/PKP utilisation and their related direct medical costs for OVCF treatment in China from the payer perspective.

**Methods:**

A population-based medical claims database of a metropolitan city in China was analysed from the payer perspective, which included all inpatient claims from 01/01/2015 to 31/12/2017. All vertebral fractures patients that met the eligibility criteria (aged ≥50 years old, having vertebral fracture diagnosis, without unrelated diseases diagnoses such as tumour and scoliosis, received PVP/PKP) were deemed as OVCF patients. Baseline characteristics, surgery rate, length of stay in hospital, time to re-surgery, and costs (including costs per hospitalisation and annual costs) were described. Survival analysis function was used to estimate the re-surgery rate.

**Results:**

Of the 50,686 patients with OVCF identified, 14,527 (28.66%) received a total number of 15,599 records of PVP/PKP surgeries from 2015 to 2017. Mean age was 75 at the first surgery captured in the database analysis period; females accounted for 79.54% of all cases. The median length of surgery stay was 9 days. Cumulative re-surgery rates were 1.22% in 30 days, 2.58% in 90 days, 3.61% in 183 days, 5.42% in 1 year, and 7.95% in 2 years. There was no significant difference in re-surgery rate between PVP and PKP (*p* = 0.3897). The median time to the re-surgery was 139 days. Mean costs per PVP/PKP-related hospitalisation were 35,906 CNY/5122 USD (34,195 CNY/4878USD for PVP, 44,414 CNY/6336 USD for PKP, *p* < 0.01). The overall costs of hospitalisation averaged 186.61 million CNY (26.62 million USD) per year in this metropolitan city.

**Conclusion:**

From 2015 to 2017, nearly one-third of OVCF inpatients received PVP/PKP and the re-surgery rate was 7.95%. PVP/PKP procedures for OVCF place a high economic burden for both the healthcare system and patients. Early detection and treatment of patients with osteoporosis are critical in China.

## Background

Osteoporosis places significant disease burden on patients, ranking the seventh among common chronic diseases with over 200 million people affected worldwide [1, 2]. In China, the prevalence of osteoporosis has been rising over the past few decades in parallel with the aging population. The prevalence of osteoporosis in mainland China was approximately 13% for adults aged 20 and over during the period of 1980–2008 and rose to about 28% for people aged 15 and over between 2012 and 2015 [3, 4] and the disease disproportionally affects females [4–6]. For most patients, osteoporosis is a silent and insidious disorder and often not diagnosed until fractures have occurred.

Osteoporotic vertebral compression fractures (OVCF) is a common and often debilitating complication of osteoporosis, leading to chronic back pain, insomnia, reduced activity, depression, and increased mortality [1]. Thirty to fifty percent of people over 50 years old could be affected by OVCF worldwide; in Europe, the incidence of OVCF was 570 per 100,000 males and 1070 per 100,000 females; in South Korea, the 5-year incidence of OVCF was 852 per 100,000 persons [7]. In Shanghai, a metropolitan city in China, 14.4% of people over 65 years old and 20.1% of people over 80 years old were suffering from OVCF and the risk of OVCF was significantly higher for females compared to their male counterparts (18.5% vs. 12.4%) [8]. Many countries around the world have studied the economic burden of OVCF. A report shows that the total annual costs of OVCF in the United States in 2005 exceeded 1 billion USD; the average annual cost of OVCF in Germany between 2006 and 2010 was 6490 EUR (7203 USD/ 50,492 CNY); the direct medical cost of OVCF patients in Canada from 2011 to 2012 was 25,965 CAD (19,993 USD/136,316 CNY) per year; China’s one-year direct medical cost for OVCF patients from 2010 to 2012 was 21,474 CNY (3063 USD) [9–12].

The OVCF treatment guidelines include both conservative and surgical interventions [1]. The conservative therapies entail initial bed rest, use of analgesics, external immobilization; while surgical treatments include percutaneous vertebroplasty (PVP) and percutaneous kyphoplasty (PKP). According to China’s guidelines, for surgical treatments, PVP is recommended for patients with OVCF who are refractory to treatment with braces and medication, but not suitable for patients with extremely severe vertebral compression fractures that cannot establish working channels and merge with the lesions of the same site requiring surgical treatment, pedicle fractures, severe compression fractures; PKP is recommended for pain or kyphosis caused by osteoporotic compression fracture, but not for stable, cured, painless osteoporotic compression fracture, osteoporotic burst fracture [1].

In addition to the surgical treatment, anti-osteoporosis treatment is an integral part of the clinical management in order to fundamentally improve bone mass and strength, and reduce the risk of re-fracture [1]. In China, the recommended anti-osteoporosis therapy includes calcium and vitamin D supplements, anti-resorptive drugs, bone anabolic drugs, and traditional Chinese medicine [13]. However, in China, a survey showed that about half of osteoporotic patients are not diagnosed even if they had fractures; moreover, less than a quarter of the patients are receiving effective anti-osteoporosis drugs before fractures [14].

Previously, a number of studies have explored the clinical efficacy and costs of PVP/PKP in China based on hospital data. Evidence showed that PVP/PKP can effectively relieve fracture-related pain, and PVP/PKP is considered as the best choice for OVCF [1, 15–17]. Nevertheless, regardless of OVCF intervention modality, PVP/PKP surgery is very expensive and can impose a heavy financial burden on patients. From 2012 to 2017, studies estimated that the hospital costs of PVP were 14,328 CNY to 44,916 CNY (about 2044 USD to 6407 USD) per time, while those of PKP were 31,681 CNY to 50,184 CNY (about 4519 USD to 7159 USD) [18–21].

However, no studies so far have examined PVP/PKP utilisation using a large-scale population-based database in China. Therefore, the primary objective of this study is to estimate PVP/PKP utilisation, including surgery rate and re-surgery rates, and related direct costs in China through analysing a citywide inpatient claims database.

## Methods

This study is a retrospective administrative claims database study to estimate the surgery/re-surgery rates and direct medical costs in patients with OVCF and receiving PVP/PKP surgery from the health payer perspective.

### Study setting and data source

Insurance claim data is a commonly used large-scale database for healthcare study. In China, over 95% of the residents have been covered by basic medical insurance. All data in this study were de-identified and extracted from an anonymous metropolitan inpatient claims database from 01/01/2015 to 31/12/2017. All OVCF patients receiving PVP/PKP surgery during such a period would be followed up to their second PVP/PKP surgery or the end of 2017 whichever comes first. The date of first surgery (PVP/PKP) during study time frame would constitute the index date. Detailed information on patients included demographics, diagnosis, treatment, costs, and so on. Patients meeting all eligibility criteria detailed below were included in this study. Because only de-identified records were used in the analysis, ethical approval and informed consent were not required, which was consistent with the local medical research policy on using electronic health data.

### Patient selection

Records with admission date from 01/01/2015 to 31/12/2017 were extracted. Inclusion criteria included aged ≥50 years old, recorded OVCF or vertebral fracture as admission diagnosis, and received PVP and/or PKP surgery. Patients diagnosed with unrelated diseases were excluded (tumour, spinal deformity, scoliosis, spondylitis, and disc herniation). Patients receiving PVP/PKP in cancer hospitals were also excluded. In the main analyses, a key assumption was that a vertebral fracture with PVP/PKP without unrelated diseases was in fact osteoporotic, which was aligned with osteoporosis diagnosis criteria according to Chinese guideline [13]. Keywords used for patient selection was displayed in Appendix A.

### Surgery and re-surgery status

All PVP/PKP surgery records were classified into three categories: PVP, PKP, and unclear. Due to lack of standardized nomination for surgery procedure, surgeries of PVP or PKP that could not be judged by information in the extracted database were marked as “unclear”. Surgery status was examined in terms of surgery rate and Length of Stay (LOS) in hospitals. Surgery rate was calculated by dividing the patient number receiving PVP/PKP by the total number of OVCF patients. Re-surgery status was explored in terms of the cumulative re-surgery rate for 3 months, 6 months, 1 year and 2 years follow-up, and median length of the time interval to re-surgery. The first surgical record captured in the database during the study period was assumed as the first surgical record of patient and ensuing PVP or PKP procedures were identified as re-surgery.

### Direct medical costs

Direct medical costs of PVP/PKP among OVCF patients were summarized in terms of both cost per hospitalisation (per visit) and annual costs (all PVP/PKP related costs in 1 year). Costs per hospitalisation were the average of total PVP/PKP surgery-related hospitalisation costs, and could be broken down to different categories, for example, medical fee, diagnosis fee, treatment fee, and so on. The annual costs were the average of the sum of all PVP/PKP surgery-related hospitalisation costs.

### Statistical methods

Descriptive analysis was employed to characterize patient profiles at their first inpatient care. Continuous variables were presented as mean, standard deviation, median, inter-quartile range whereas categorical variables were displayed in frequencies and percentages. A re-surgery function was developed to illustrate the probability of re-surgery with the time interval between the first and second surgery. Patients without re-surgery were censored at the end of 2017. Kaplan-Meier method was used to establish the re-surgery function. Based on the function, cumulative re-surgery rates and their 95% confidence intervals were estimated at different time points, specifically, 3 months, 6 months, 1 year, and 2 years. The calculation of the time interval to re-surgery is shown in the following formula:

$$ Time\ inteval= admission\ date\ of\ 2 nd\  visit- discharge\ date\ of\ 1 st\  visit $$

Non-parametric statistical tests were used for the costs, days (Mann-Whitney Test for two groups, and Kruskal-Wallis Test for three or more groups) and re-surgery rate (Log-rank Test and Wilcoxon Test) comparison, α = 0.05 was used as the significant level for all comparisons.

All analyses were conducted using Stata SE 14.

### Subgroup analyses

Two subgroup analyses were performed on age and sex groups because they have a significant effect on the incidence of osteoporosis and vertebral fractures. Sex was divided into two categories: male and female; age was divided into five categories: 50–59, 60–69, 70–79, 80–89, 90+. LOS, re-surgery rates and costs were counted and compared between subgroups.

### Sensitivity analysis

A key assumption of the main analysis was that all patients who met the eligibility criteria were identified as OVCF patient even if they did not have an OVCF diagnosis. To test the robustness of the results, a sensitivity analysis was performed focusing on patients who had established OVCF diagnosis. All the statistical analyses in the main analysis were implemented in the sensitivity analysis, except for the annual costs.

## Results

### Study population

**Fig. 1:**
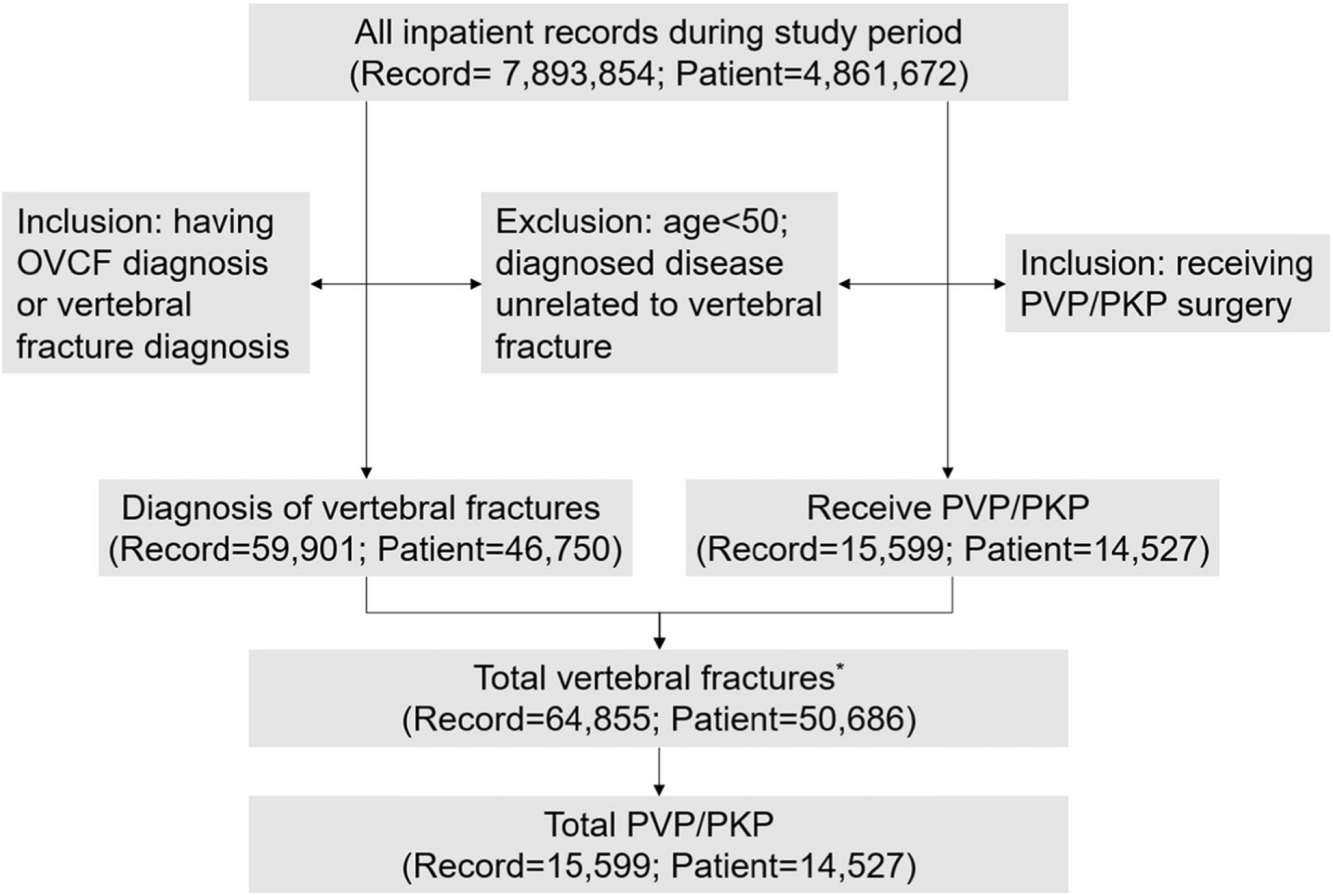
Patient selection process among those undergoing PVP/PKP between Jan 1, 2015 and Dec 31, 2017. *All vertebral fractures patients who meet inclusion/exclusion criteria are deemed as OVCF; the number of OVCF patients enrolled is less than the sum of patients undergoing surgery and those diagnosed with vertebral fractures because of overlap between the two groups

Extracted raw data included 8,524,679 records. A total of 50,686 OVCF patients with 64,855 hospitalisations were identified, among them 14,527 (28.66%) patients received PVP/PKP with 15,599 records (Fig. 1).

### Baseline characteristics

Of the 50,686 OVCF patients, 15,437 (30.46%) were male and 35,249 (69.54%) were female. Among 14,527 patients, 8760 (60.30%) underwent PVP, 3048 (20.98%) underwent PKP, and 2719 (18.72%) were unclear for the first surgery. PVP/PKP patients were 75-year-old on average and females accounted for 79.54%. Most surgeries were performed in general hospitals (60.69%) and Chinese traditional medicine hospitals (28.22%). Tertiary care hospitals accounted for 92.16%. (Table 1).

**Table 1:** Baseline characteristics for PVP/PKP patients

	Total Population		PVP		PKP		Unclear	
N	14,527		8760		3048		2719	
**Age**								
Mean	75.10		75.08		75.48		74.73	
Standard Deviation	9.35		9.34		9.35		9.37	
Median	76		76		77		76	
**Age group (n, %)**								
50 to < 60	825	5.68%	474	5.41%	176	5.77%	175	6.44%
60 to < 70	3336	22.96%	2084	23.79%	629	20.64%	623	22.91%
70 to < 80	5094	35.07%	3014	34.41%	1082	35.50%	998	36.70%
80 to < 90	4659	32.07%	2820	32.19%	1021	33.50%	818	30.08%
> =90	613	4.22%	368	4.20%	140	4.59%	105	3.86%
**Gender (n, %)**								
Male	2972	20.46%	1801	20.56%	651	21.36%	520	19.12%
Female	11,555	79.54%	6959	79.44%	2397	78.64%	2199	80.88%
**Hospital Category (n, %)**								
General Hospital	8816	60.69%	5313	60.65%	1654	54.27%	1849	68.00%
TCM Hospital	4100	28.22%	2868	32.74%	534	17.52%	698	25.67%
Orthopaedic Hospital	856	5.89%	173	1.97%	643	21.10%	40	1.47%
ITCWM Hospital	703	4.84%	362	4.13%	216	7.09%	125	4.60%
MCC Hospital	30	0.21%	23	0.26%	0	0.00%	7	0.26%
Other Hospitals	22	0.15%	21	0.24%	1	0.03%	0	0.00%
**Hospital Level (n, %)**								
Tertiary	13,388	92.16%	8003	91.36%	2728	89.50%	2657	97.72%
Secondary	1081	7.44%	724	8.26%	314	10.30%	43	1.58%
Primary	48	0.33%	30	0.34%	6	0.20%	12	0.44%
Unknown	10	0.07%	3	0.03%	0	0.00%	7	0.26%
**Admission Year (n, %)**								
2015	4105	28.26%	1467	16.75%	629	20.64%	2009	73.89%
2016	5799	39.92%	3926	44.82%	1387	45.51%	486	17.87%
2017	4623	31.82%	3367	38.44%	1032	33.86%	224	8.24%

Abbreviation: *TCM* Traditional Chinese Medicine, *ITCWM* Integrated Traditional Chinese and Western Medicine, *MCC* Maternity and Child Care

Note: Unclear means patient underwent PVP/PKP but could not distinguish the type of surgery

### Surgery and re-surgery status

Of all OVCF patients, the total surgery rate was 28.66%, of which PVP was 17.30% and PKP was 6.03% (Table 2). Median LOS in the hospital was 9 days, of which PVP was 9 days and PKP was 10 days (Table 3).

**Table 2:** PVP/PKP surgery rate for patient diagnosed as OVCF

	Total	PVP	PKP	Unclear
The number of vertebral fracture patient*	50,686	–	–	–
The number of PVP/PKP patient	14,527	8767	3057	2703
The proportion of PVP/PKP among those having vertebral fracture	28.66%	17.30%	6.03%	5.33%

Note: *All vertebral fractures patients who meet inclusion/exclusion criteria are considered as OVCF

**Table 3:** Length of stay for patient receiving PVP/PKP (day)

	Total Population	PVP	PKP	Unclear
N	15,599	3277	9477	2845
Mean	10.66	11.74	10.43	10.15
Standard Deviation	8.06	8.54	8.25	6.63
Minimum	1	1	1	1
1st Quartile	6	7	6	6
Median	9	10	9	9
3rd Quartile	13	14	13	12
Maximum	256	191	256	92

**Fig. 2:**
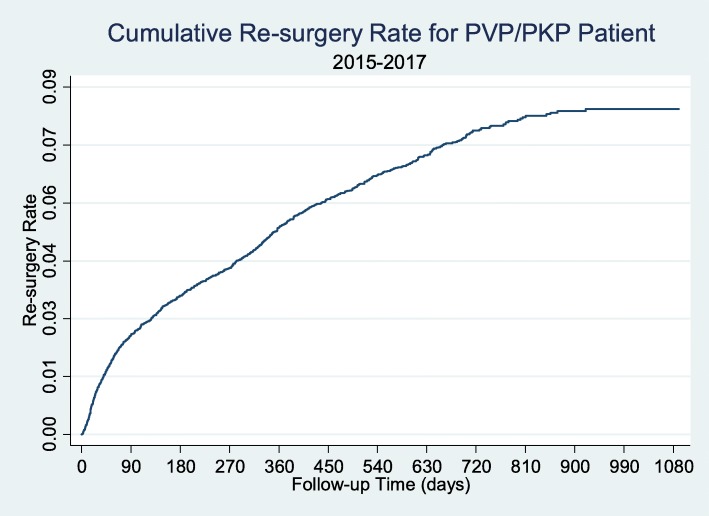
Re-surgery Rate for PVP/PKP Patient, by Kaplan-Meier Curve

**Fig. 3:**
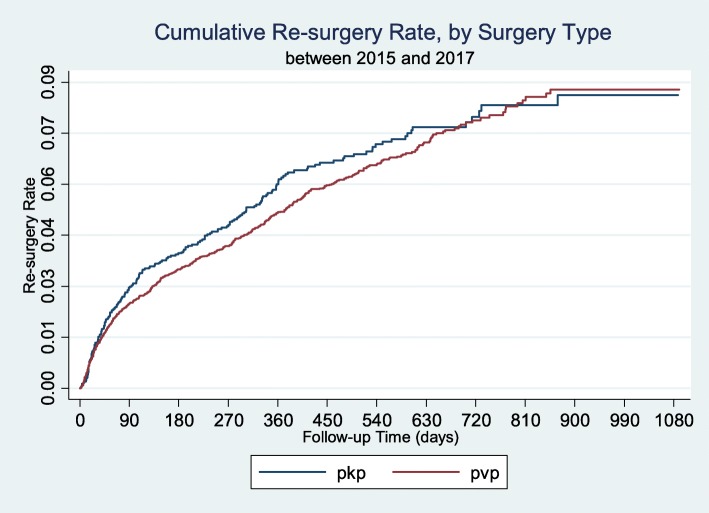
Re-surgery Rate for PVP/PKP Patient respectively, by Kaplan-Meier Curve

Among all the patients during the study period, the re-surgery rate was 1.22% in 30 days, 2.58% in 90 days, 3.61% in 183 days, 5.42% in 1 year, and 7.95% in 2 years (Table 4; Figs. 2 and 3). Median time interval to re-surgery was 139 days (Table 5). There was no significant difference in the re-surgery rate between PVP and PKP, regardless of the type of statistical tests (Log-rank: *p* = 0.3897; Wilcoxon: *p* = 0.1829).

**Table 4:** Re-surgery rate for patient receiving PVP/PKP (by patient)

Time	Total Population			PVP			PKP			Unclear		
	Re-surgery rate	SE	95% CI	Re-surgery rate	SE	95% CI	Re-surgery rate	SE	95% CI	Re-surgery rate	SE	95% CI
30 days	0.0122	0.0009	(0.0105, 0.0141)	0.0124	0.0012	(0.0103, 0.0149)	0.0135	0.0021	(0.0099, 0.0182)	0.0103	0.0019	(0.0071, 0.0149)
90 days	0.0258	0.0013	(0.0233, 0.0285)	0.0249	0.0017	(0.0218, 0.0284)	0.0296	0.0031	(0.0241, 0.0362)	0.0243	0.0030	(0.0192, 0.0309)
183 days	0.0361	0.0016	(0.0332, 0.0393)	0.0352	0.0020	(0.0315, 0.0393)	0.0397	0.0036	(0.0333, 0.0473)	0.0352	0.0036	(0.0289, 0.0429)
365 days	0.0542	0.0020	(0.0504, 0.0582)	0.0520	0.0025	(0.0472, 0.0572)	0.0615	0.0046	(0.0530, 0.0712)	0.0528	0.0043	(0.0530, 0.0712)
730 days	0.0795	0.0027	(0.0743, 0.0850)	0.0796	0.0038	(0.0723, 0.0875)	0.0833	0.0064	(0.0716, 0.0968)	0.0770	0.0053	(0.0672 0.0881)
1090 days	0.0896	0.0033	(0.0834, 0.0963)	0.0946	0.0051	(0.0851, 0.1051)	0.0945	0.0077	(0.0805, 0.1109)	0.0798	0.0056	(0.0695, 0.0916)

Abbreviation: *SE* Standard Error, *CI* Confidence Interval

**Table 5:** Time interval to re-surgery for patient receiving more than once PVP/PKP

	Total Population	PVP	PKP	Unclear
N	916	516	200	200
Mean	211.383	202.7035	181.54	263.62
Standard Deviation	203.591	200.0735	181.4055	224.165
Minimum	0	0	0	2
1st Quartile	43	38	39	59
Median	139	131	107	194
3rd Quartile	338	328	294	428
Maximum	920	856	869	920

### Direct medical costs

The annual total costs attributable to PVP/PKP related hospitalisation from 2015 to 2017 were 153 million CNY (22 million USD), 178 million CNY (25 million USD), and 229 million CNY (33 million USD), respectively. The overall costs of hospitalisation were 187 million CNY (27 million USD) per year in this anonymised metropolitan city of China (Table 6).

**Table 6:** Annual costs for PVP/PKP patients (CNY)

	2015	2016	2017	Mean
**Annual costs**	152,759,120	178,167,264	228,915,056	186,613,813
**Annual visits**	4250	5109	6240	–

Total costs per PVP/PKP hospitalisation averaged 35,906 CNY (5122 USD) (Table 7). The total costs of PKP hospitalisation were significantly higher than that of PKP [34,195 CNY (4878 USD) for PVP, 44,414 CNY (6336 USD) for PKP, *P* < 0.01] (Table 8).

**Table 7:** The distribution of costs for PVP/PKP patients per hospitalisation (CNY)

Costs Classification	N	Mean	S.D	Minimum	1st Quartile	Median	3rd Quartile	Maximum
**Comprehensive Medical Services**								
General Medical Service	14,333	561	712	0	276	427	654	24,743
General Treatment Operation	14,332	325	1072	0	71	136	266	47,826
Nursing	14,775	184	285	0	72	118	209	11,840
**Diagnosis**								
Laboratory Diagnosis	14,631	1221	1097	0	732	1007	1360	44,375
Imaging Diagnosis	14,456	2083	1191	0	1268	1938	2641	13,672
Clinical Diagnosis	14,332	157	520	0	26	45	133	22,454
**Treatment**								
Non-operative Treatment	14,456	548	2421	0	0	60	332	78,637
Operative Treatment	15,235	2721	2230	0	1825	1920	2755	72,731
*Anesthetic Fee*^a^	15,234	308	693	0	25	55	143	7953
*Surgery Fee*^a^	15,282	2234	1164	0	1800	1800	2340	28,000
**Medicine**								
Western Medicine Fee	15,597	3883	4673	0	1434	2848	4939	180,000
*Antibiotics Fee*^a^	14,332	211	1497	0	0	0	21	104,000
Chinese Patent Medicine Fee	15,266	512	941	0	0	123	630	19,004
Chinese Herbal Medicine Fee	15,091	70	179	0	0	0	51	5693
**Materials**								
Disposable Medical Materials for Surgery	NA	22,980	NA	NA	NA	NA	NA	NA
Disposable Medical Materials for Treatment	14,864	1320	4891	0	20	118	380	82,753
**Total Costs**	15,592	35,906	19,601	1000	24,042	33,416	42,079	365,000
*Out-of-pocket Costs*^a^	14,652	17,752	16,885	0	5944	13,533	25,674	213,000

Abbreviation: *S. D* Standard Deviation

Note: ^a^ indicates secondary subject costs; Cost of Disposable Medical Materials for Surgery category: PVP data gained from chart review; PKP and Total data of this category inferred from the 64%*Total Cost

**Table 8:** Average costs for PVP/PKP patients per hospitalisation (CNY), by surgery group

Costs Classification	PVP		PKP		***P*** value
	Mean	S.D	Mean	S.D	
**Comprehensive Medical Services**					
General Medical Service	548	696	602	690	< 0.01
General Treatment Operation	350	1266	262	620	< 0.01
Nursing	189	319	187	226	< 0.01
**Diagnosis**					
Laboratory Diagnosis	1245	1123	1164	1129	< 0.01
Imaging Diagnosis	2054	1201	2224	1146	< 0.01
Clinical Diagnosis	167	553	141	461	< 0.01
**Treatment**					
Non-operative Treatment	433	2010	633	1593	< 0.01
Operative Treatment	2783	2578	2823	1596	< 0.01
*Anesthetic Fee*^a^	232	577	580	927	< 0.01
*Surgery Fee*^a^	2281	1278	2184	979	< 0.01
**Medicine**					
Western Medicine Fee	3737	4648	4383	5250	< 0.01
*Antibiotics Fee*^a^	197	1461	274	1918	< 0.01
Chinese Patent Medicine Fee	513	980	510	906	0.69
Chinese Herbal Medicine Fee	67	178	82	182	0.04
**Materials**					
Disposable Medical Materials for Surgery	21,885	NA	28,425	NA	NA
Disposable Medical Materials for Treatment	1424	4904	1686	6108	0.03
**Total Costs**	34,195	20,115	44,414	17,287	< 0.01
*Out-of-pocket Costs*^a^	16,183	16,102	19,187	18,526	< 0.01

Abbreviation: *S. D* Standard Deviation

Note: ^a^indicates secondary subject costs; Cost of Disposable Medical Materials for Surgery category: PVP data gained from chart review; PKP and Total data of this category inferred from the 64%*Total Cost; the method used for difference between groups is Mann-Whitney Test

### Subgroup analyses

Median LOS was 9 days for both male and female. However, the statistical test showed that the difference in LOS distribution between male and female was significant (*p* < 0.01). Median LOS was 8,8,9,10,10 for each age groups, respectively. The difference among age groups was also statistically significant (p < 0.01). (Supplementary Table 1).

The 2-year cumulative re-surgery rates were 6.92% for male and 8.20% for female, respectively. Patients in the 50–59 age group had the lowest 2-year cumulative re-surgery rate (4.48%), while patients in 80–89 age groups had the highest (10.22%). The statistical difference was not significant (*p* = 0.192) between sex but significant among age groups (*p* < 0.01). (Supplementary Table 2).

The average hospital costs of the male were significantly higher than that of female [37,950CNY (5414USD) vs 35,383CNY (5048USD), p < 0.01]. There were also significant differences in the average costs of hospitalisation for different age groups (p < 0.01), with patients in 50–59 age group having the highest costs [37,970CNY (5417USD)] and patients in 60–69 age group having the lowest [35,221CNY (5024USD)]. (Supplementary Table 3 and 4).

### Sensitivity analysis

Patients with exact OVCF diagnosis were included in the sensitivity analysis. 18,567 OVCF patients with 23,056 visits were identified, among them 6237 (33.59%) patients received PVP/PKP with 6538 records (Supplementary Table 5). Of these patients undergoing surgery, 1086 (17.41%) were male and 5151 (82.59%) were female; they were 75-year-old on average.

Median LOS in hospitals was 9 days, of which PVP was 8 days and PKP was 10 days (Supplementary Table 6). The re-surgery rate was 0.93% in 30 days, 2.10% in 90 days, 2.75% in 183 days, 3.63% in 1 year, and 5.51% in 2 years Supplementary Table 7). There was no difference in re-surgery rate between the PVP and PKP (Supplementary Table 8). The median time interval to re-surgery was 97 days (Supplementary Table 9). Total costs per hospitalisation averaged 34,561 CNY (4930 USD) (Supplementary Table 10).

In addition, outliers of costs and time interval were jointly checked. In 14,527 patients, 28 patients had time interval to re-surgery less than 7 days; only 2 of them had total costs less than 13,842CNY/1975USD (the 5th percentile of total costs distribution); 13 of them had total costs less than 33,416CNY/4767USD (the median of total costs). Re-surgery and first surgery of these patients can be considered as one treatment. However, due to the very small patient number, it does not affect the overall results.

## Discussion

This study was a retrospective longitudinal study aiming at measuring the burden of PVP/PKP due to OVCF in China from the payer perspective. To our knowledge, our study was the first to estimate the utilisation of PVP/PKP procedures and their costs for OVCF treatment in China by analysing real-world city-wide claims data. We noted the utilisation of PVP/PKP was high for OVCF patients with 28.66% PVP/PKP surgery rate and 7.95% 2-year cumulative re-surgery rate. Median LOS in hospitals was 9 days, and median time interval to re-surgery was 139 days. Hospitalisation costs due to PVP/PKP were high: per hospitalisation cost averaged 35,906 CNY (5122 USD), and annual hospitalisation costs in the metropolitan city totalled 187 million CNY (27 million USD). It brought a significant burden to both medical insurance institution and patients.

Subgroup analyses and sensitivity analysis indicated that men had shorter hospital stays but higher hospital costs than women. In addition, there was no difference in reoperation rates between men and women. With the increase of age, both LOS and the re-surgery rates increased (the sudden drop in the re-surgery rate in 90+ age group might be due to the impact of sample size, mortality, etc.). However, the costs of hospitalisation for the 50–59 age group were the highest, which emphasized the value of early intervention for osteoporosis.

Overall, the results of this study were comparable with those of previous studies. In this study, the number of female OVCF patients was about 2.3 times that of male patients, which is consistent with the higher risk of OVCF in females in other studies [1, 4, 8, 22]. The surgery rate of PVP/PKP in OVCF patients in our study was also similar to the finding of another study at 23.1% [23]. A comparison of re-surgery rate among studies was not conducted due to the lack of studies on PVP/PKP re-surgery rates.

Whether it is PVP or PKP, surgery-related inpatient costs are high. In contrast, taking a short-term view, PVP seems to be better than PKP because there was no significant difference in the re-surgery rate between PVP and PKP, but the costs of PVP were nearly 30% lower than that of PKP. Another clinical study in China also recommended PVP because the clinical result (pain relief) differed little but the costs of PKP were higher [24]. However, due to the lack of confounder control in this study, we should be more cautious about asserting a causal relationship between surgery types and the outcomes. The costs associated with vertebral fractures in many countries are well documented: in America, the costs per surgery-related hospitalisation were 7805 USD (54,713 CNY) for PVP and 12,032 USD (84,344 CNY) for PKP in 2006, 9837 USD (68,957 CNY) for PVP and 13,187 USD (92,441 CNY) for PKP in 2007 and 2008 [25, 26]. The results of US studies are similar to our study, where the costs of PKP hospitalisation were higher than PVP. However, it is worth noting that in terms of long-term costs, the results are reversed. In America, the two-year cumulative costs were 44,496 USD (311,917 CNY) for PVP and 41,339 USD (289,786 CNY) for PKP between 2006 and 2010 [27]. In Germany, patients’ four-year cumulative costs were 42,510 EUR (330,775 CNY/47,186USD) for PVP and 39,014 EUR (303,529 CNY/43,306USD) for PKP between 2006 and 2010 [28]. One reason is that patients with PKP surgery use fewer drugs and pay lower maintenance costs after surgery. PKP significantly reduced 6.8–7.9% treatment costs during the 2-year post-surgery periods in America and reduced 33% painkiller costs during the 4-year post-surgery periods in German [27, 28]. The surgical sequelae and its burden would be an interesting point for future research.

Aside from surgery intervention, conservative treatment for OVCF is widely employed in China. Conservative treatment is recommended for mild/chronic OVCF, while severe/acute OVCF is treated by surgery [29]. Anticoagulant therapy of low molecular weight heparin calcium injection can be given during bed rest; non-steroidal anti-inflammatory analgesics are mainly used to relieve acute pain; anti-osteoporosis medication, such as alendronate sodium, and complex calcium carbonate vitamin D tablets can also be employed [29, 30]. In terms of treatment effect, for pain relief, the short-term effect of surgery is better than that of conservative treatment, but no difference was observed in terms of long-term effect [31–35]. Recently, a study indicated that conservative treatment with anabolic drug could achieve comparative outcomes than PVP/PKP in treating acute OVCF [36]. However, from the perspective of recovery of vertebral stability and vertebral height, surgery is superior to conservative treatment [34].

Although successful PVP/PKP treatment for OVCF can effectively alleviate pains and other symptoms, the procedures are not free of untoward effects. Studies have explored that the PVP/PKP may accelerate local bone absorption due to bone cement, thereby increasing the risk of recurrent fracture of the surgical vertebra [37–40]. In addition, studies also reported that additional stress of adjacent vertebrae caused by the cement augmentation and cement leakage are important factors in causing new adjacent vertebral fractures after PVP/PKP [41–44].

To avoid OVCF, anti-osteoporosis therapy should be considered as primary prevention. Osteoporosis and resulting osteoporotic vertebral fractures typically develop silently with a long-time window from the initial decrease in bone density to the occurrence of OVCF. Use of anti-osteoporosis drugs can reduce the risk of fractures. For women with osteoporosis but without vertebral fractures, alendronate significantly reduced the first vertebral fracture by 44% [45]. Studies have shown that anti-osteoporosis treatment after fracture can reduce the risk of re-fracture by 40% within 3 years [46]. In this regard, anti-osteoporosis treatment should be considered as part of long-term treatment strategies to reduce the risk of fracture [47–50]. Although anti-osteoporosis therapy plays a significant role in OVCF prevention, the current situation of drug use is not optimistic. This might be due to the low diagnosis rate of osteoporosis and vertebral fractures. What’s more, there is a large gap in the diagnosis and prevention of osteoporosis and vertebral fractures among different level hospitals, especially in community hospitals where the diagnosis ability is poor. Although inadequate diagnosis and prevention of osteoporosis is consensus, related real-world studies are lacking. The insufficiency of osteoporosis diagnosis and prevention can only be indirectly understood from the situation of fracture patients. A study in mainland China showed only 13.9% of patients used anti-osteoporosis drugs before fractures [23]. Moreover, a retrospective study showed that in China, the rate of misdiagnosis of vertebral fractures was 54.27%; while in the patients with vertebral fractures diagnosis, 61.33% were definitely diagnosed as osteoporosis or OVCF, and only 28% were given anti-osteoporosis therapy [51]. Compounding the problem is poor compliance with the medications as short-term medication intake has no evidentiary clinical benefits for fracture prevention [52–54]. In China, an analysis of medical insurance claims database from 2009 to 2010 showed that the adherence to bisphosphonate treatment was even worse with the mean Medication Possession Ratio (MPR) being 0.34, 0.22, and 0.15 at the 3rd, 6th, and 12th month over the follow-up period, respectively; moreover, only 2.1% patients were observed with high adherence (MPR > 0.8) during the 12-month follow-up [55].

The strength of our study stems from a well-defined study population which was population-based, i.e. residents of the metropolitan city covered by government health insurance were all included. The findings are likely to be more robust than those derived from a single hospital or hospitals from convenient sampling. Nevertheless, our study also has a number of limitations. On the one hand, some of the shortcomings of this study are due to the limitations of the database. Mortality and comorbidity are important in health research. However, our study was based on administrative claim database which was unable to support research about survival of patients in real world. Besides, claim database lacks detailed medical information in terms of comorbidity. In addition, researchers should be cautious about extrapolating findings to other regions or cities in China or to patients without health insurance because of variation in adopting surgical intervention for OVCF, health insurance coverage schemes and steep costs for self-paying patients. On the other hand, study design can lead to some estimation bias. First, we assumed all vertebral fracture cases meeting eligibility criteria were osteoporotic. This may include a few patients with non-osteoporotic vertebral fractures. In order to justify the assumption, we ran the sensitivity analysis among patients who had established OVCF diagnosis. The results were in line with those in the main analysis, which proves that our assumption is reliable. Second, there may be omissions in selecting PVP/PKP patients due to the lack of standardized procedure name, which would lead to the under-estimation of the surgery rate. Third, re-surgery rate calculation could also be underestimated due to the lack of knowledge of patients’ history before 2015. In addition, because we only captured the records of hospitalisations during which surgery was performed, there are two circumstances of postoperative patient care: discharged at the same visit until full recovery; discharged shortly after surgery, home or transferred to other hospitals for postoperative care. In the latter case, since we were not able to make the linkage between surgery and rehabilitative stay, the LOS and costs of hospitalisation may be underestimated.

## Conclusion

From 2015 to 2017, about a third of OVCF patients received PVP/PKP surgery and the 2-year cumulative re-surgery rate reached 7.95%. PVP/PKP due to OVCF brought a high economic burden on China’s healthcare system. Early detection and treatment of patients with osteoporosis to prevent OVCF are critical in China.

### Supplementary information

**Additional file 1.**

## Data Availability

The datasets generated during and/or analysed during the current study are not publicly available due to government policy but are available from the corresponding author on reasonable request.

## Appendix A: Keyword used in patient selection

### Keywords used in inclusion criteria (Chinese)

Vertebral fracture diagnosis: “骨折” and “椎”

OVCF diagnosis: (“骨质疏松” or “骨松”) and (“骨折” and “椎”).

PVP/PKP: including (“PVP” or “PKP” or “pvp” or “pkp”) or (“经皮” and”椎体”) or 3(“椎”and”成形”and”成型”) and excluding [“活检”or “椎板” or (“动脉” and “成形”) or (“血管” and “成形”) or (“动脉” and “成型”) or (“血管” and “成型”) or “减压成形术” or “椎管” or “支架” or “摘除术” or “消融” or “内固定” or “复位术” or “侧弯成形” or “封闭术” or “扩大成形” or “纤维环成形” or “髓核成形” or “臭氧成形” or “单开门成形”].

### Keywords used in exclusion criteria

Tumour: “瘤”

Cancer hospital: “肿瘤医院”.

Spinal deformity and scoliosis: “脊柱”and (“畸形” or “侧弯” or “侧凸” or”后凸”).

Spondylitis: “脊柱炎”.

Disc herniation: “椎间盘”and (“突出” or “脱出”)

## Abbreviations

OVCF: Osteoporotic vertebral compression fractures
PVP: Percutaneous vertebroplasty
PKP: Percutaneous kyphoplasty
LOS: Length of stay
