# Supplementary material for: Resources utilisation and economic burden of percutaneous vertebroplasty or percutaneous kyphoplasty for treatment of osteoporotic vertebral compression fractures in China: a retrospective claim database study
Source: BMC Musculoskelet Disord. 2020 Apr 17;21:255. doi: 10.1186/s12891-020-03279-1 (PMC7165412; doi:10.1186/s12891-020-03279-1)
Supplement: Supplementary file 1 — Additional file 1. [file 12891_2020_3279_MOESM1_ESM.docx]

**Supplementary Table 1 Subgroup analysis: Length of stay for patient receiving PVP/PKP (day), sex and age subgroups**

| **Subgroup** | | **N** | **Mean** | **S.D** | **Minimum** | **1st Quartile** | **Median** | **3rd Quartile** | **Maximum** | **P value** |
| --- | --- | --- | --- | --- | --- | --- | --- | --- | --- | --- |
| Sex | Male | 3,175 | 11.38 | 9.166 | 1 | 6 | 9 | 14 | 191 | <0.01 |
|  | Female | 12,424 | 10.47 | 7.745 | 1 | 6 | 9 | 13 | 256 |  |
| Age | 50-59 | 847 | 10.36 | 7.78 | 1 | 6 | 8 | 13 | 92 | <0.01 |
|  | 60-69 | 3,471 | 9.76 | 7.87 | 1 | 6 | 8 | 12 | 191 |  |
|  | 70-79 | 5,483 | 10.46 | 7.84 | 1 | 6 | 9 | 13 | 256 |  |
|  | 80-89 | 5,140 | 11.34 | 8.24 | 1 | 7 | 10 | 14 | 206 |  |
|  | 90+ | 658 | 12.01 | 9.23 | 1 | 7 | 10 | 14 | 124 |  |

Abbreviation: S.D - Standard Deviation

Note: Mann-Whitney Test for two groups comparison, and Kruskal-Wallis Test for three or more groups comparison

**Supplementary Table 2 Subgroup analysis: Re-surgery rate for patient receiving PVP/PKP, sex and age subgroups**

| Subgroup | | Time | | | | | P value |
| --- | --- | --- | --- | --- | --- | --- | --- |
|  |  | 30 days | 90 days | 183 days | 365 days | 730 days |  |
| Sex | Male | 0.0108 | 0.0237 | 0.0344 | 0.0542 | 0.0692 | 0.192 |
|  | Female | 0.0126 | 0.0263 | 0.0366 | 0.0542 | 0.0820 |  |
| Age | 50-59 | 0.0024 | 0.0061 | 0.0151 | 0.0212 | 0.0448 | <0.01 |
|  | 60-69 | 0.0051 | 0.0129 | 0.0218 | 0.0311 | 0.0470 |  |
|  | 70-79 | 0.0134 | 0.0264 | 0.0384 | 0.0573 | 0.0871 |  |
|  | 80-89 | 0.0181 | 0.0375 | 0.0479 | 0.0740 | 0.1022 |  |
|  | 90+ | 0.0098 | 0.0278 | 0.0345 | 0.0473 | 0.0642 |  |

**Supplementary Table 3 Subgroup analysis: Average costs for PVP/PKP patients per hospitalisation (CNY), by sex group**

| **Costs Classification** | **Male** | | **Female** |  | **P value** |
| --- | --- | --- | --- | --- | --- |
|  | **Mean** | **S.D** | **Mean** | **S.D** |  |
| **Comprehensive Medical Services** |  |  |  |  |  |
| General Medical Service | 620 | 735 | 546 | 705 | <0.01 |
| General Treatment Operation | 417 | 1,062 | 301 | 1,073 | <0.01 |
| Nursing | 215 | 400 | 175 | 246 | <0.01 |
| **Diagnosis** |  |  |  |  |  |
| Laboratory Diagnosis | 1,360 | 1,587 | 1,185 | 927 | <0.01 |
| Imaging Diagnosis | 2,129 | 1,309 | 2,071 | 1,158 | 0.996 |
| Clinical Diagnosis | 202 | 802 | 145 | 417 | <0.01 |
| **Treatment** |  |  |  |  |  |
| Non-operative Treatment | 570 | 2,473 | 543 | 2,407 | 0.51 |
| Operative Treatment | 2,788 | 2,619 | 2,704 | 2,119 | 0.62 |
| *Anesthetic Fee ** | 313 | 707 | 306 | 690 | 0.61 |
| *Surgery Fee ** | 2,257 | 1,165 | 2,228 | 1,164 | 0.96 |
| **Medicine** |  |  |  |  |  |
| Western Medicine Fee | 4,326 | 6,593 | 3,770 | 4,031 | 0.09 |
| *Antibiotics Fee ** | 384 | 2,540 | 167 | 1,073 | <0.01 |
| Chinese Patent Medicine Fee | 516 | 968 | 510 | 935 | 0.11 |
| Chinese Herbal Medicine Fee | 74 | 216 | 69 | 169 | 0.49 |
| **Materials** |  |  |  |  |  |
| Disposable Medical Materials for Surgery | NA | NA | NA | NA | NA |
| Disposable Medical Materials for Treatment | 1,316 | 4,997 | 1,320 | 4,864 | 0.11 |
| **Total Costs** | 37,950 | 22,968 | 35,383 | 18,608 | <0.01 |
| *Out-of-pocket Costs** | 17,888 | 18,703 | 17,717 | 16,384 | 0.04 |
| Abbreviation: S.D - Standard Deviation | | | | | |
| Note: * indicates secondary subject costs; Disposable Medical Materials for Surgery is not available in database; The method used for difference between groups is Mann-Whitney Test. | | | | | |

**Supplementary Table 4 Subgroup analysis: Average costs for PVP/PKP patients per hospitalisation (CNY), by age group**

| **Costs Classification** | **50-59** | | **60-69** | | **70-79** | | **80-89** | | **90+** | | **P value** |
| --- | --- | --- | --- | --- | --- | --- | --- | --- | --- | --- | --- |
|  | **Mean** | **S.D** | **Mean** | **S.D** | **Mean** | **S.D** | **Mean** | **S.D** | **Mean** | **S.D** |  |
| **Comprehensive Medical Services** |  |  |  |  |  |  |  |  |  |  |  |
| General Medical Service | 543 | 588 | 508 | 604 | 543 | 729 | 606 | 768 | 655 | 747 | <0.01 |
| General Treatment Operation | 312 | 1,009 | 279 | 1,225 | 309 | 1,118 | 359 | 901 | 442 | 1,143 | <0.01 |
| Nursing | 167 | 274 | 159 | 219 | 177 | 264 | 201 | 256 | 258 | 697 | <0.01 |
| **Diagnosis** |  |  |  |  |  |  |  |  |  |  |  |
| Laboratory Diagnosis | 1,201 | 1,497 | 1,120 | 835 | 1,180 | 944 | 1,310 | 1,253 | 1,430 | 1,450 | <0.01 |
| Imaging Diagnosis | 2,006 | 1,406 | 1,961 | 1,209 | 2,066 | 1,165 | 2,174 | 1,172 | 2,247 | 1,066 | <0.01 |
| Clinical Diagnosis | 168 | 691 | 130 | 330 | 136 | 408 | 182 | 593 | 250 | 1,031 | <0.01 |
| **Treatment** |  |  |  |  |  |  |  |  |  |  |  |
| Non-operative Treatment | 486 | 1,922 | 436 | 1,924 | 624 | 2,886 | 546 | 2,261 | 606 | 2,325 | 0.11 |
| Operative Treatment | 3,029 | 2,560 | 2,861 | 2,410 | 2,721 | 2,310 | 2,613 | 2,032 | 2,435 | 1,320 | <0.01 |
| *Anesthetic Fee ** | 444 | 926 | 338 | 745 | 302 | 680 | 282 | 638 | 223 | 541 | <0.01 |
| *Surgery Fee ** | 2,501 | 1,808 | 2,324 | 1,326 | 2,236 | 1,198 | 2,148 | 875 | 2,065 | 674 | <0.01 |
| **Medicine** |  |  |  |  |  |  |  |  |  |  |  |
| Western Medicine Fee | 3,942 | 6,709 | 3,524 | 4,290 | 3,706 | 4,013 | 4,220 | 4,828 | 4,541 | 6,653 | <0.01 |
| *Antibiotics Fee ** | 376 | 4,016 | 203 | 1,778 | 160 | 734 | 235 | 1,120 | 272 | 1,103 | 0.02 |
| Chinese Patent Medicine Fee | 500 | 853 | 465 | 784 | 511 | 952 | 538 | 1,021 | 562 | 1,046 | <0.01 |
| Chinese Herbal Medicine Fee | 56 | 147 | 67 | 160 | 69 | 166 | 77 | 208 | 67 | 169 | 0.03 |
| **Materials** |  |  |  |  |  |  |  |  |  |  |  |
| Disposable Medical Materials for Surgery | NA | NA | NA | NA | NA | NA | NA | NA | NA | NA | NA |
| Disposable Medical Materials for Treatment | 1,634 | 5,106 | 1,149 | 4,304 | 1,159 | 4,553 | 1,494 | 5,388 | 1,745 | 5,861 | <0.01 |
| **Total Costs** | 37,970 | 24,579 | 35,221 | 20,173 | 35,222 | 19,185 | 36,674 | 18,414 | 36,557 | 21,295 | <0.01 |
| *Out-of-pocket Costs** | 22,330 | 20,807 | 20,005 | 18,336 | 17,966 | 16,870 | 15,529 | 14,738 | 15,513 | 16,221 | <0.01 |
| Abbreviation: S.D - Standard Deviation | | | | | | | | | | | |
| Note: * indicates secondary subject costs; Disposable Medical Materials for Surgery is not available in database; The method used for difference between groups is Kruskal-Wallis Test | | | | | | | | | | | |

**Supplementary Table 5 Sensitivity analysis: PVP/PKP surgery rate for patient with confirmed OVCF diagnosis**

|  | Total | PVP | PKP | Unclear |
| --- | --- | --- | --- | --- |
| The number of OVCF patient | 18,567 | - | - | - |
| The number of PVP/PKP patient | 6,237 | 3,522 | 1,488 | 1,227 |
| The proportion of PVP/PKP among OCVF patients | 33.59% | 18.97% | 8.01% | 6.61% |

**Supplementary Table 6 Sensitivity analysis: Length of stay for patient with confirmed OVCF diagnosis and receiving PVP/PKP (day)**

|  | **Total Population** | **PVP** | **PKP** | **Unclear** |
| --- | --- | --- | --- | --- |
| N | 6,538 | 3,715 | 1,560 | 1,263 |
| Mean | 10.37 | 9.91 | 11.81 | 9.92 |
| Standard Deviation | 7.15 | 7.22 | 7.43 | 6.33 |
| Minimum | 1 | 1 | 1 | 1 |
| 1st Quartile | 6 | 6 | 7 | 6 |
| Median | 9 | 8 | 10 | 9 |
| 3rd Quartile | 13 | 12 | 14 | 12 |
| Maximum | 206 | 206 | 79 | 71 |

**Supplementary Table 7 Sensitivity analysis: Re-surgery rate for patient with confirmed OVCF diagnosis and receiving PVP/PKP**

| **Time** | **Total Population** | **PVP** | **PKP** | **Unclear** |
| --- | --- | --- | --- | --- |
| 30 days | 0.0093 | 0.0103 | 0.0101 | 0.0057 |
| 90 days | 0.0210 | 0.0215 | 0.0235 | 0.0173 |
| 183 days | 0.0275 | 0.0284 | 0.0291 | 0.0231 |
| 1 year | 0.0363 | 0.0361 | 0.0384 | 0.0344 |
| 2 years | 0.0551 | 0.0556 | 0.0593 | 0.0518 |

**Supplementary Table 8 Sensitivity analysis: Statistical tests of PVP/PKP re-surgery rate for patient with confirmed OVCF diagnosis**

| **Test Method** | **Chi^2^** | **P value** |
| --- | --- | --- |
| **Log-rank** | 0.1 | 0.7544 |
| **Wilcoxon** | 0.16 | 0.6923 |

**Supplementary Table 9 Sensitivity analysis: Time interval to re-surgery for patient with confirmed OVCF diagnosis and receiving more than once PVP/PKP**

|  | **Total Population** | **PVP** | **PKP** | **Unclear** |
| --- | --- | --- | --- | --- |
| N | 269 | 141 | 68 | 60 |
| Mean | 193.33 | 172.05 | 179.01 | 259.55 |
| Standard Deviation | 202.13 | 189.57 | 196.53 | 225.12 |
| Minimum | 0 | 3 | 0 | 13 |
| 1st Quartile | 36 | 30 | 33 | 67 |
| Median | 97 | 76 | 85 | 191 |
| 3rd Quartile | 322 | 266 | 291 | 432 |
| Maximum | 934 | 775 | 726 | 934 |

**Supplementary Table 10 Sensitivity analysis: The distribution of costs for PVP/PKP patients with OVCF diagnosis per hospitalisation (CNY)**

| **Costs Classification** | **N** | **Mean** | **S.D** | **Minimum** | **1st Quartile** | **Median** | **3rd Quartile** | **Maximum** |
| --- | --- | --- | --- | --- | --- | --- | --- | --- |
| **Comprehensive Medical Services** | | | | | | | | |
| General Medical Service | 5,931 | 548 | 665 | 0 | 276 | 424 | 644 | 22,375 |
| General Treatment Operation | 5,930 | 259 | 686 | 0 | 64 | 129 | 247 | 19,780 |
| Nursing | 6,141 | 179 | 215 | 0 | 72 | 119 | 212 | 3,476 |
| **Diagnosis** | | | | | | | | |
| Laboratory Diagnosis | 6,096 | 1,152 | 861 | 0 | 724 | 989 | 1,305 | 31,935 |
| Imaging Diagnosis | 5,979 | 2,085 | 1,111 | 0 | 1,310 | 1,989 | 2,695 | 13,672 |
| Clinical Diagnosis | 5,930 | 130 | 350 | 0 | 26 | 45 | 115 | 12,814 |
| **Treatment** | | | | | | | | |
| Non-operative Treatment | 5,979 | 541 | 1,874 | 0 | 0 | 72 | 395 | 48,277 |
| Operative Treatment | 6,405 | 2,731 | 1,906 | 0 | 1,825 | 1,958 | 2,794 | 36,366 |
| *Anesthetic Fee ** | 6,404 | 350 | 736 | 0 | 25 | 55 | 180 | 7,953 |
| *Surgery Fee ** | 6,413 | 2,190 | 997 | 0 | 1,800 | 1,800 | 2,340 | 28,000 |
| **Medicine** | | | | | | | | |
| Western Medicine Fee | 6,536 | 3,753 | 3,747 | 0 | 1,525 | 2,901 | 4,923 | 74,643 |
| *Antibiotics Fee ** | 5,930 | 160 | 694 | 0 | 0 | 0 | 25 | 25,213 |
| Chinese Patent Medicine Fee | 6,364 | 477 | 855 | 0 | 0 | 130 | 578 | 17,495 |
| Chinese Herbal Medicine Fee | 6,314 | 66 | 162 | 0 | 0 | 0 | 44 | 2,717 |
| **Materials** | | | | | | | | |
| Disposable Medical Materials for Surgery | NA | NA | NA | NA | NA | NA | NA | NA |
| Disposable Medical Materials for Treatment | 6,211 | 1,141 | 4,495 | 0 | 33 | 120 | 372 | 64,788 |
| **Total Costs** | 6,533 | 34,561 | 18,056 | 1,000 | 23,521 | 32,690 | 41,326 | 265,925 |
| *Out-of-pocket Costs** | 6,094 | 17,549 | 15,686 | 0 | 6,235 | 13,693 | 25,429 | 192,000 |
| Abbreviation: S.D - Standard Deviation | | | | | | | | |
| Note: * indicates secondary subject costs; Disposable Medical Materials for Surgery is not available in database | | | | | | | | |
